# Personal Goals, User Engagement, and Meal Adherence within a Personalised AI-Based Mobile Application for Nutrition and Physical Activity

**DOI:** 10.3390/life14101238

**Published:** 2024-09-27

**Authors:** Elena Patra, Anna Kokkinopoulou, Saskia Wilson-Barnes, Kathryn Hart, Lazaros P. Gymnopoulos, Dorothea Tsatsou, Vassilios Solachidis, Kosmas Dimitropoulos, Konstantinos Rouskas, Anagnostis Argiriou, Elena Lalama, Marta Csanalosi, Andreas F. H. Pfeiffer, Véronique Cornelissen, Elise Decorte, Sofia Balula Dias, Yannis Oikonomidis, José María Botana, Riccardo Leoni, Duncan Russell, Eugenio Mantovani, Milena Aleksić, Boris Brkić, Maria Hassapidou, Ioannis Pagkalos

**Affiliations:** 1 Nutrition Information Systems Laboratory (NISLAB), Department of Nutritional Sciences and Dietetics, International Hellenic University, 57400 Thessaloniki, Greece; 2 School of Biosciences, Faculty of Health and Medical Sciences, University of Surrey, Guildford GU2 7WG, UK; 3 Centre for Research & Technology Hellas, 57001 Thessaloniki, Greece; 4 Department of Endocrinology and Metabolic Diseases, Charité-Universitätsmedizin Berlin, 10117 Berlin, Germany; 5 Department of Rehabilitation Sciences, KU Leuven, 3001 Leuven, Belgium; 6 Interdisciplinary Centre for the Study of Human Performance (CIPER), Faculdade de Motricidade Humana, Universidade de Lisboa, 1499-002 Lisbon, Portugal; 7 Intrasoft International S.A., 55535 Thessaloniki, Greece; 8 CGI Information Systems and Management Consultants S.A., 28050 Madrid, Spain; 9 Datawizard, 00138 Rome, Italy; 10 Ocado Group, Hatfield, London AL10 9UL, UK; 11 Research Group on Law, Science, Technology and Society, Faculty of Law & Criminology, Vrije Universiteit Brussel, 1050 Brussels, Belgium; 12 Research and Development Institute for Information Technology in Biosystems, BioSense Institute, 21000 Novi Sad, Serbia

**Keywords:** personalised nutrition, mobile health, AI-based personalisation, food choice drivers

## Abstract

Mobile applications have been shown to be an effective and feasible intervention medium for improving healthy food intake in different target groups. As part of the PeRsOnalized nutriTion for hEalthy livINg (PROTEIN) European Union H2020 project, the PROTEIN mobile application was developed as an end-user environment, aiming to facilitate healthier lifestyles through artificial intelligence (AI)-based personalised dietary and physical activity recommendations. Recommendations were generated by an AI advisor for different user groups, combining users’ personal information and preferences with a custom knowledge-based system developed by experts to create personalised, evidence-based nutrition and activity plans. The PROTEIN app was piloted across different user groups in five European countries (Belgium, Germany, Greece, Portugal, and the United Kingdom). Data from the PROTEIN app’s user database (*n* = 579) and the PROTEIN end-user questionnaire (*n* = 446) were analysed using the chi-square test of independence to identify associations between personal goals, meal recommendations, and meal adherence among different gender, age, and user groups. The results indicate that weight loss-related goals are more prevalent, as well as more engaging, across all users. Health- and physical activity-related goals are key for increased meal adherence, with further differentiation evident between age and user groups. Congruency between user groups and their respective goals is also important for increased meal adherence. Our study outcomes, and the overall research framework created by the PROTEIN project, can be used to inform the future development of nutrition mobile applications and enable researchers and application designers/developers to better address personalisation for specific user groups, with a focus on user intent, as well as in-app features.

## 1. Introduction

The core aim of mobile health (mHealth) applications (apps) is to enable users’ transition to health-related behaviours, such as improving eating habits and promoting a healthy lifestyle for disease prevention, by using behavioural change techniques [1]. mHealth apps have been shown to be an effective and feasible intervention tool for improving healthy food intake for different target groups [2,3,4]. The significant consumer reach of smartphone devices offers a great opportunity to develop effective smartphone solutions that will improve diet quality and encourage healthy eating behaviours and lifestyles [5].

However, mHealth apps are often subject to dropout rates or loss in original user engagement and adherence to recommendations [6,7]. Research has shown that up to 80% of all participants in mHealth interventions only engage at a minimum level, do not log into the mHealth app more than once, and do not consistently use the app in the long term [7]. In addition, studies examining user engagement found that as few as 3.9% of participants used the mHealth apps for >15 days [8]. Minimising participant dropout rates in mHealth studies or with commercial apps is crucial for many reasons. Digital health studies can suffer from the introduction of bias, weak study power, and low effectiveness. Thus, the main advantages of accessibility and reach to diverse samples in terms of race, ethnicity, gender, age, and education level are reduced, and therefore their potential positive impact is minimised [9]. Understanding the factors that influence users’ acceptance and use of mHealth apps will help to improve users’ actual usage behaviour [10].

There are many parameters that are known to promote user attrition or retention, which are either intrinsic/functional to apps (i.e., in-app features and technical details) or user-/patient-related, such as niche population groups or gender. Examples of intrinsic characteristics that support engagement are perceived user-friendliness or ease of use [10,11,12], technical stability and performance [6,13], feedback mechanisms [14], social support, push notifications or reminders [15], in-app support from users or experts, and many more. Parameters of compensation in pay-for participation models have also been mentioned but are more interesting within research initiatives [15]. On the other hand, technical difficulties, such as poor user experience (UX) design, are often reported as elements that increase user attrition. More strategic elements include, but are not limited to, purposeful development for specific populations, personalisation, and customisation [6].

Personalisation is a crucial factor to increase the UX and effectiveness of mHealth solutions, and it can enhance adherence to treatment by tailoring interventions to individual needs and preferences [16]. When it comes to user-related parameters, demographic characteristics and personal motivations are known to influence attrition and engagement in different ways. More specifically, age, type of population group, personal goals and motivations, and prior experience with mHealth apps are some factors that influence user acceptance and engagement [11,17,18,19,20]. Although the usage of mHealth applications is rapidly increasing, studies from users’ perspectives are limited [19]. User engagement is critical to the success of mHealth apps and respective interventions. Previous research has highlighted the need for clear understanding of users’ health needs and the introduction of apps with a high degree of novelty [11]. Therefore, it is important to understand which parameters are the most impactful and influence attrition and engagement across various user groups.

The success of mHealth applications depends on sustained user engagement, which is critical in achieving desired health outcomes. Research indicates that personal goals play a pivotal role in fostering this engagement. According to Locke and Latham’s Goal-Setting Theory, specific and challenging goals can enhance motivation and performance by providing clear targets and feedback mechanisms [20]. In the context of mHealth, personalised goals such as daily step counts, weight loss targets, medication adherence schedules, and stress reduction activities can act as intrinsic motivators, aligning with Self-Determination Theory, which posits that autonomy, competence, and relatedness are fundamental in maintaining engagement [21]. Studies have shown that users are more likely to remain engaged with health apps when they can set and track personal goals, which in turn supports fulfilment of their autonomy and competence [22,23]. For instance, a randomised controlled trial on a mobile app for physical activity found that participants who set specific goals like walking a certain number of steps per day or increasing their weekly exercise duration demonstrated significantly higher levels of physical activity and app usage compared to those who did not set goals [24]. Additionally, goal-setting features that provide regular feedback and allow for goal adjustment are crucial in maintaining user motivation and preventing disengagement due to perceived failure or lack of progress [25]. As such, the incorporation of personal goal-setting mechanisms within mHealth applications—whether they are focused on fitness, dietary habits, mental health, or chronic disease management—is not only beneficial but essential in promoting long-term user engagement and achieving meaningful health outcomes.

Based on the above, and taking into consideration the opportunities and challenges within an mHealth ecosystem, this research was focused on (i) identifying personal goals associated with user engagement and meal adherence within users of an AI-based mHealth app and (ii) exploring any gender, age, and user group differences related to the above.

## 2. Materials and Methods

### 2.1. The PROTEIN Mobile App

Within the framework of the European Union-funded H2020 project “PeRsOnalized nutriTion for hEalthy living” (PROTEIN) [11,26,27], the PROTEIN mobile app was developed as an end-user environment for the PROTEIN ecosystem, aiming to provide a novel artificial intelligence (AI)-personalised nutritional advisor, generating daily and weekly meal plans. The PROTEIN app (available on Google Play Store, https://buff.ly/2Qz3M7i, accessed on 26 September 2024) took into consideration the following: (a) user profiles (e.g., physical characteristics, dietary choices, health conditions, and preferences), (b) nutrition and physical activity experts’ recommendations on macro- and micro-nutrient intake, and (c) a database of meal plans developed by nutrition experts for varying population groups (see Data Availability Statement). Through a series of fuzzy rules and filtering mechanisms, PROTEIN’s nutritional AI advisor selected suitable meals from a pool of the expert-defined available meals. To achieve this, the advisor first filtered meals based on explicit user preferences and health conditions (e.g., gluten-free products for users with allergies and low-energy or low-fat meals for users with excess weight/obesity). The suitable meals were then aggregated to form 24 h meal plans, which were further re-adapted according to users’ engagement and feedback through meal confirmations or rejections and meal rating. The details of the knowledge-based recommendation framework (PROTEIN AI Advisor), including the expert knowledge base and the reasoning-based AI decision support system (NAct: The Nutrition & Activity Ontology for Healthy Living), are described in detail elsewhere [27,28,29].

Upon registration, users were asked to provide their demographic and anthropometric information (including weight and height), dietary preferences, needs or medical conditions, and level of physical activity. Based on the above, each user (with the help of a registered nutritionist for Groups B and C) was linked to one or more personal goals from an extensive list (Table S1 in Supplementary Materials, File A). Following this process, the AI advisor began recommending tailored Nutrition and Physical Activity (NAP) plans to the user. During app usage, each user had the option to rate their liking of meals and physical activities recommendations, as well as to confirm or reject them, allowing the AI advisor to constantly re-adapt recommendations every 2 weeks to further increase personalisation.

### 2.2. The PROTEIN App Pilots

The PROTEIN app was piloted in five European countries (i.e., Belgium, Germany, Greece, Portugal, and the United Kingdom) across three different user groups, namely: Group A—users with no health conditions, normal BMI, non-exercisers; Group B—users under nutritionist supervision (individuals with overweight, athletes, and leisure exercisers); and Group C—users under medical and nutritional supervision (people with obesity, type 2 diabetes (T2D), cardiovascular disease (CVD), poor-quality diets, and certain deficiencies). The subgroups were supervised by their corresponding piloting partner(s) in one or more countries. Common inclusion criteria across all groups required participants to be (i) Android users, (ii) able to provide written consent, (iii) able to read/write, (iv) ≥18 years of age, and (v) not regular user of another nutrition support mobile application. Exclusion criteria included people with (i) eating disorders, (ii) mental health issues, (iii) drug abuse, and (iv) pregnancy. Further details on each pilot-specific inclusion and exclusion criterion (as self-reported by users) are available under Table S3 in Supplementary Materials.

All PROTEIN pilot partners contributed data through their respective pilots, which started at different times between May and June 2022. For the purposes of this research, data were extracted on a common date across pilots in October 2022.

Following the end of the pilots, participants were invited to complete the PROTEIN end-user questionnaire either via an external link or directly through the PROTEIN app (see Supplementary Materials—PROTEIN questionnaire). The questionnaire evaluated the PROTEIN app’s effectiveness in helping users meet their set goals (e.g., NAP variety, engagement and adherence, personalisation features, and motivation), overall experience with the app environment (e.g., interface interaction, easiness to use, and effectiveness), and technical aspects (e.g., speed, unexpected behaviour, security, and privacy).

### 2.3. Data Sources and Analysis Principles

The PROTEIN pilots resulted in an extensive list of datasets (e.g., user data, sensor data, and app usage data), which can be used to extract useful and innovative data to better understand and evaluate the impact of an mHealth ecosystem in achieving personalised nutrition. To interpret users’ choices for this study, two specific datasets were used, namely: (i) data from the PROTEIN database (i.e., user and meal-choice data from the use of the app), and (ii) answers from the PROTEIN end-user questionnaire.

The two sources were intentionally disconnected for anonymisation, data, and privacy protection purposes (i.e., PROTEIN database users could not be linked to questionnaire respondents). Additionally, as the completion of the PROTEIN end-user questionnaire was optional, the number of responses was different to the PROTEIN database’s user sample size. As such, both sources had to be treated separately in terms of their analysis but were analysed based on the same principles: identifying personal goals associated with user engagement and meal adherence and exploring any gender, age, and user group differences for the above.

### 2.4. Data Analysis

**Figure 1:**
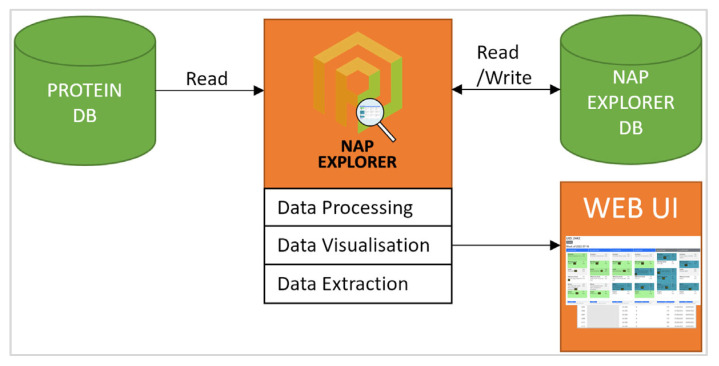
Τhe PROTEIN NAP Explorer Framework. A visual representation of the Web User Interface (WEB UI) architecture, providing searchable user insights based on PROTEIN database data.

In order to extract, visualise, and analyse data from the secure PROTEIN database, a prototype web-based tool was developed by project partners (the “NAP Explorer”) (see Figure 1). The NAP Explorer parsed user data and presented them in a Web interface, as well as performing the data transformation actions necessary for further processing and analysis. The “NAP Explorer” tool created downloadable text export (CSV) and/or Excel files for any PROTEIN user(s), using data from the PROTEIN database. This dataset provided a unique insight into user behaviour, as it not only included meal information generated by the AI advisor but also users’ interactions with them (meal confirmations/rejections of recommended meals). Filtered data from the NAP dashboard view were downloaded directly through the browser (CSV) text export. The chi-square goodness-of-fit test was performed to determine whether variables were equally distributed across users. Fisher’s exact test was performed on the 2 × 2 tables that were observed for (i) personal goals × gender, (ii) personal goals × age, and (iii) personal goals × user groups, to determine any statistically significant associations between the respective categorical variables. Significance was set at a 95% confidence level (*p* < 0.05) for all analysis. All statistical analyses were performed using XLSTAT 2024 (Addinsoft, Paris, France).

Analysis of the PROTEIN end-user questionnaire was performed on pooled data from all participating pilots and further analysed for gender, age, and user group differences. The data used for the purposes of this study were categorical (ordinal or nominal) and were treated using non-parametric statistical procedures. The chi-square goodness-of-fit test was performed to determine whether variables were equally distributed across users. Fisher’s exact test was performed on the 2 × 2 tables that were observed for (i) goals × gender, (ii) goals × age, and (iii) goals × user groups, to determine any statistically significant associations between the respective categorical variables. Correspondence analysis (CA) was run on the contingency tables between groups, and the CA biplot was generated as a technique to visualise the relationships between the respective variable groups. Significance was set at a 95% confidence level (*p* < 0.05). All statistical analyses were performed using XLSTAT 2024 (Addinsoft, Paris, France).

## 3. Results

### 3.1. User Demographics

After removing any “inactive” users (i.e., those with no confirmation or rating activities), 579 users were included in the analysis through the NAP Explorer. Participants were mostly women (56%), while most users were between 25 and 45 years old (53%). The most prevalent user group included in this analysis was Group A (49.9%), followed by athletes and leisure exercisers (16.9%), and individuals with overweight (13.3%) (Table 1).

A total of 512 users across all pilots responded to the PROTEIN end-user questionnaire. Following the removal of incomplete responses, the final dataset included responses from 446 individuals. Respondents were mainly female (*n* = 282, 63.2%) and aged between 25 and 44 years old (*n* = 263, 58.9%). Most respondents were users without a specific health condition, with a normal BMI, and who were non-exercisers (Group A) (28.9%) and people with overweight (28%), followed by people with poor-quality diets (18.6%), athletes and leisure exercisers (11.7%), people with obesity (4.7%), iron-deficiency anaemia (2.9%), CVD (2%), and T2D (2%) (Table 1).

### 3.2. Personal Goals and Motivations

**Figure 2:**
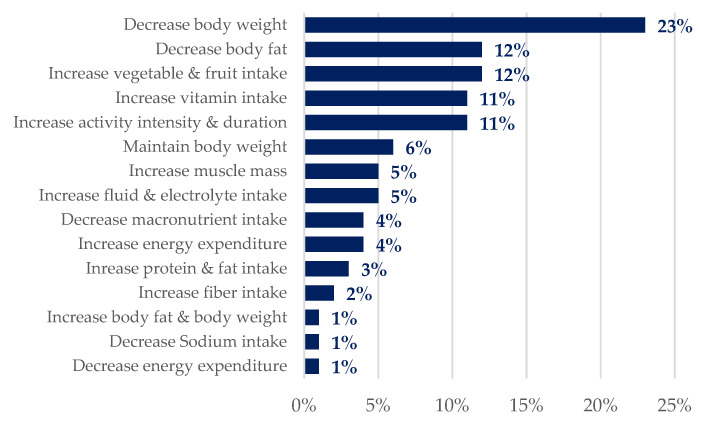
Ranked distribution (percentages in total) of users assigned a personal goal when first signing into the PROTEIN app (more than one goal could apply).

When looking at the overall goal distribution through the PROTEIN database, the most prevalent goals were weight-related, more specifically, “decrease body weight” (23%) and “decrease body fat” (12%), followed by “increase vegetable & fruit intake”, “increase vitamin intake”, and “increase activity intensity & duration” (11%) (Figure 2).

**Figure 3:**
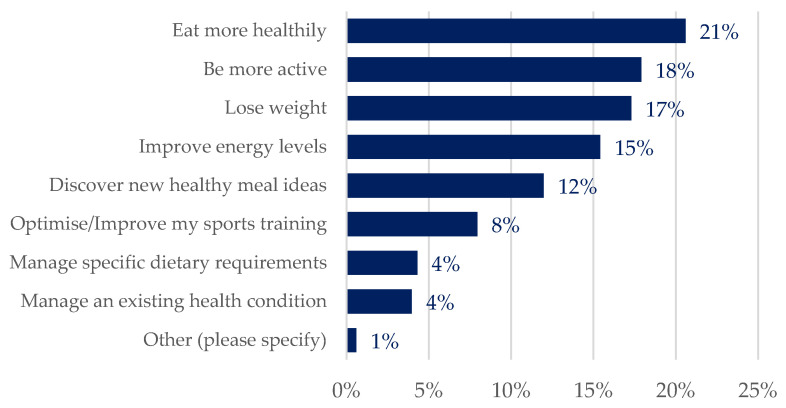
Ranked distribution (percentages in total) of self-reported goals as selected by users in the PROTEIN end-user questionnaire (more than one goal could apply).

The most common health and wellbeing goals as reported by users were to “eat more healthy” (21%), “be more active” (18%), and “lose weight” (17%). The items “optimize/improve sports training” (8%), “manage a specific dietary requirement”, and “manage an existing health condition” (4% for both) were the least common across all respondents (Figure 3).

#### Gender, Age, and User Group Associations

When evaluating the goal distribution based on gender, no significant differences were found. Men and women equally prioritised the same goals. To specify, “eat more healthy” (21% women vs. 20% men), “be more active” (18% women vs. 17% men), “lose weight” (18% women vs. 16% men) and “improve energy levels” (16% women vs. 14% men) were their main goals (Table S4 in Supplementary Materials).

Pairwise comparisons between age groups and personal user goals show that “lose weight” was more common amongst users aged 55–64 years old (26%, *p* = 0.027) and “manage an existing health condition” was more often selected by respondents over 65 years old (10%, *p* = 0.035). Meanwhile, “optimize/improve sports training” was more prevalent within the younger demographic (18–24 years) (14%, *p* = 0.008) (Table S5 in Supplementary Materials).

When it comes to user groups, statistically significant associations were identified for all cases except people with poor-quality diets and iron-deficiency anaemia. The goal “losing weight” was the most common response for people with obesity (31%, *p* = 0.006) and for people with overweight (23%, *p* < 0.001). Conversely, “optimize/improve sports training” was selected more often by athletes/leisure exercisers (17%, *p* < 0.001), while “managing an existing health condition” was more commonly reported by people with obesity (11%, *p* = 0.016) and people with CVD (15%, *p* = 0.014) and T2D (19%, *p* = 0.012) than other user groups. Furthermore, “managing specific dietary requirements” was also a more common goal amongst people with T2D (19%, *p* = 0.014) than other user groups (Table S6 in Supplementary Materials).

### 3.3. User Engagement and Meal Adherence

Overall meal confirmation activity (i.e., setting a meal as “confirmed” or “rejected”, which can be used as a proxy for user engagement) was higher across weight-related goals. Furthermore, meal confirmations were statistically more common when the goal was one of the following: “decrease body fat”, “maintain body weight”, “decrease carbohydrate intake”, “increase activity intensity”, “increase body weight”, “decrease energy expenditure”, and “decrease sodium intake” (all *p*-values < 0.001) (Table S7 in Supplementary Materials).

**Figure 4:**
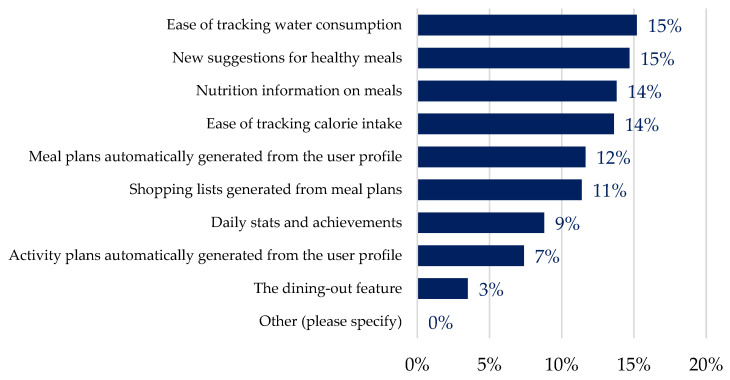
Ranked distribution (percentage in total) of app features supporting meal adherence (more than one answer could apply).

Meal confirmations occurred more often (78%) than rejections (22%), suggesting successful personalisation to individual user profiles. With respect to app features that facilitated meal adherence, the most useful ones, as reported by all users, were perceived to be “ease of tracking water consumption” (15%) and “new suggestions for healthy meals” (15%), followed by “ease of tracking calorie intake” (14%) and “nutrition information on meals” (14%) (Figure 4). Conversely, the “dining out feature” (3%) was rated as the least helpful aspect of the app; however, it was only tested within a small subgroup of users. No significant differences were found for gender, while, when looking at age differences, “activity plans automatically generated from the PROTEIN app” were considered statistically more helpful by participants aged 18–24 years (*p* = 0.006) and statistically least helpful by those aged 55–64 years (*p* = 0.028).

#### Gender, Age, and User Group Goal Associations

**Figure 5:**
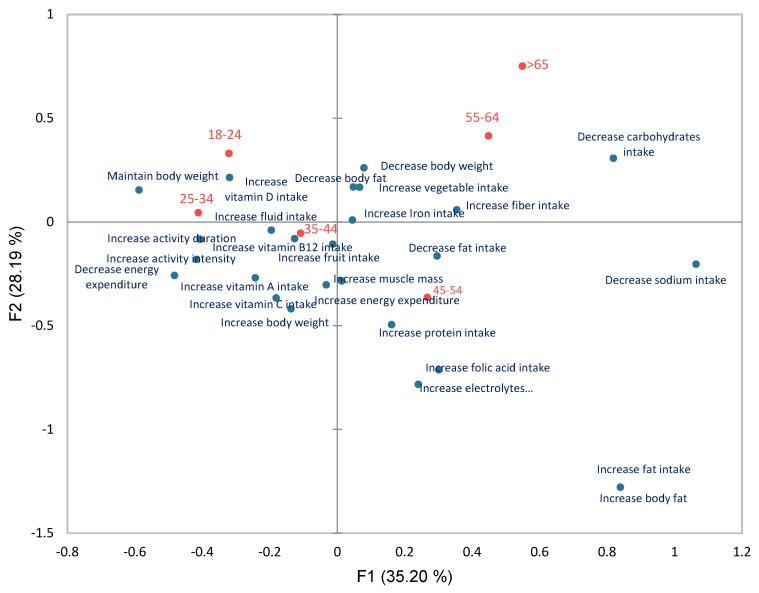
Correspondence analysis biplot for age groups and personal goals (63.40% of total variance explained). The biplot visualises the relative relationships between and within age groups and personal goals (based on the respective contingency table) based on their principal coordinates.

**Figure 6:**
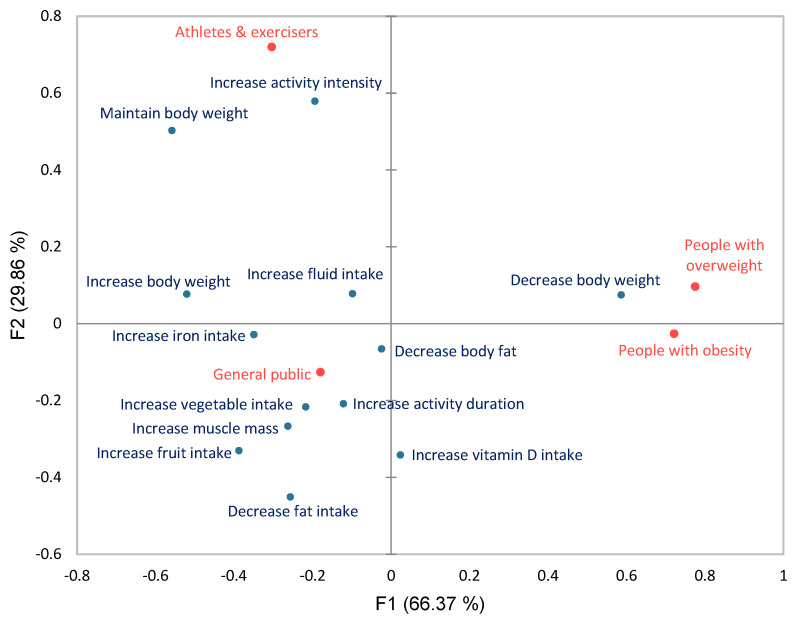
Correspondence analysis biplot for user groups and personal goals (96.23% of total variance explained). The biplot visualises the relative relationships between and within user groups and personal goals (based on the respective contingency table) based on their principal coordinates.

This section presents results only within meal confirmation activity, to identify associations of personal goals with gender, age and user group in meal adherence. The full test statistics can be found in Supplementary Materials Tables S8–S10. Additionally, correspondence analysis (CA) was run to visualise goal associations within meal confirmation activity for age and user groups (Figure 5 and Figure 6).

With respect to gender, women were more likely to confirm that they had consumed meals regardless of their goals (all *p*-values < 0.001 and 0.013). Meanwhile, the incidences of meal confirmation were more common for men when their goals were set to “increase energy expenditure”, “increase fruit intake”, “increase activity duration”, “increase activity intensity”, “maintain body weight”, and “decrease body fat” (all *p*-values < 0.001) (Table S8 in Supplementary Materials).

Meal adherence across all age groups was higher when goals were set at “decrease fat intake”, “decrease carbohydrate intake”, and “decrease body fat”, as these were associated with the most age groups at higher-than-expected proportions (all *p*-values < 0.001) (Table S9 in Supplementary Materials). Furthermore, similar age groups shared more goal associations between each other, indicating that adherence is more similar.

More specifically, “increase vitamin B12 intake” and “increase activity intensity” were associated with both the 18–24 and 25–24 age groups (all *p*-values < 0.001). “Increase iron intake” was uniquely associated with the 18–24 age group, while “decrease energy expenditure” was uniquely associated with the 25–34 age group (all *p*-values < 0.001). “Decrease body fat” was associated with both the 25–34 and 35–44 age groups, and with 55–64 and >65 (both *p*-values < 0.001). Overall, the 35–44 and 45–54 age groups shared the most goal associations; “increase body weight”, “increase folic acid intake”, and “decrease fat intake” all led to more common confirmations for both age groups (all *p*-values < 0.001). “Increase vitamin A intake” was uniquely associated with the 35–44 age group (all *p*-values < 0.001). “Decrease sodium intake”, “decrease fat intake”, and “decrease carbohydrates intake” were all associated with the 45–54 and 55–64 age groups (all *p*-values < 0.001). “Increase protein intake” and “increase electrolyte intake” were uniquely associated with the 45–54 age group, while “increase fibre intake” was uniquely associated with the 55–64 age group (all *p*-values < 0.001). Finally, the most associations were found for the 18–24 age group, indicating the highest adherence for a group across the set goals (all *p*-values < 0.001) (Table S10 in Supplementary Materials).

Since certain user groups prioritised only a single goal or very few goals (e.g., people with T2D and people with iron-deficiency anaemia), in order to avoid data skewing, results under this section are presented only for user groups that shared the most goals and meal confirmations. More specifically, these were the (i) users with no health conditions and a normal BMI and non-exercisers, (ii) people with overweight, (iii) people with obesity, and (iv) athletes and leisure exercisers. Based on the results, more associations were found for Group A (all *p*-values < 0.001). People with overweight confirmed meals more often when their goal was set to “decrease body weight” (*p* < 0.001), whereas people with obesity confirmed meals more commonly when their goals were set to “increase activity duration” and “decrease body weight” (*p*-value < 0.001). Finally, for athletes and exercisers, “increase body weight”, “maintain body weight”, “increase iron intake”, “increase activity intensity”, and “increase fluid intake” were goals associated with more frequent meal confirmations (*p*-value < 0.001) (Table S10 in Supplementary Materials).

## 4. Discussion

The present research focused on personal goals driving user engagement and meal adherence in an AI-enabled personalised nutrition and physical activity app. The analyses carried out on the PROTEIN end-user questionnaire feedback focused on self-reported goals and app features that facilitated meal adherence, while analysis of the PROTEIN database enabled a closer look into individual user engagement, based primarily on meal confirmations. The influence of age, gender, and user group was investigated in both cases.

Most PROTEIN users were young, female, and without any specific health condition, had a normal BMI, and were non-exercisers. Previous research on users’ perspectives on mHealth apps also showed that active users tended to be female [19]. Across all active users, weight loss was the primary goal driving increased user engagement and adherence to meal recommendations, followed by health-related and physical activity goals. A recent cross-sectional survey across mHealth users also identified weight loss and physical activity as key reasons for the adoption and use of mHealth apps [30].

Our study identified further differentiation among age and user groups. Specifically, weight loss was more prevalent among people with overweight and people with obesity than other user groups. A recent systematic review focusing on user engagement with mHealth apps concluded that increased app user engagement may be associated with improved weight and body mass index [18]. Our study found that health-related goals were more closely associated with people with obesity, CVD, and T2D. This finding is in line with previous research on people with obesity who were found to use app functions for achieving health behaviour goals [31], as well as people with T2D whose motives were better glucose control or a reduced number of medications, rather than weight management or any other goal [32]. When relevant features such as the “dining out” and “shopping cart” were made available to users (Group A), they were evaluated as being useful.

With regard to the suggestion adherence among engaged users, meal confirmations occurred more than rejections (78% vs. 22%, respectively), indicating a base level of successful personalisation by the AI advisor to individual users. Although goal setting did not significantly differ by gender, as also shown in previous studies [31,33,34], women were overall more likely to follow meal recommendations across their set goals, except for physical activity-related ones, where men were more adherent to app recommendations. Similar results have also been reported in other studies. For instance, a study reviewing adolescent usage and attrition on SidekickHealth (an mHealth social app health game with nutrition, mental health, and physical activity elements) found that male participants in the intervention group (*n* = 61) were active for significantly longer than female participants (*n* = 56) (29.155 vs. 20.433 days; x^2^ _1_ = 6.574; *p* < 0.001) [35].

With respect to age group associations, weight loss and health were important goals for meal adherence within older age groups (55–64 and >65, respectively). This is in line with previous research addressing the role of age in mHealth usage. A study exploring perceived benefits and barriers in using mHealth apps among Korean adults >50 years old (*n* = 323) found that participants who perceived mHealth apps as useful for self-monitoring, obtaining tailored health information, or maintaining healthy lifestyles were more likely to use them than those who did not [31]. Another study focusing on barriers and motivations in using mHealth apps among African men who completed an online survey (*n* = 311) reported that older men were more likely to participate in mHealth interventions if they would help them better understand or self-manage a disease and add to their overall quality of life [17]. On the other hand, meal adherence among younger users was associated with physical activity-related goals. An exploratory study addressing the socio-demographic and individual characteristics of users who utilised specific mHealth app functions found younger age associated only with a higher usage of weight-related ones [19].

Meal adherence among specific user groups was increased when there was congruency between user groups and their respective personal goals. More specifically, athletes and leisure exercisers followed the meal recommendations more often when their goals were related to physical activity. This is in line with research focusing on indicators of retention in digital health studies. A study combining results from digital health studies across 10,000 participants found that, among other factors, having the condition of interest was significantly associated with retention among users [36]. Goal setting is recognised as a key factor influencing engagement and behaviour change [37], with plenty of evidence suggesting that goals should be defined by users rather than imposed on them [38,39,40,41].

Beyond personal goals and motivations, our study found that certain in-app features drove increased engagement within some age or user groups. Increased user engagement and adherence is known to be impacted by in-app features [6], and conversely, a lack of desired in-app features is associated with abandonment [30]. The results from our study show that within available PROTEIN features, the ease of tracking water and calorie consumption, new meal suggestions, and meal information were the most helpful features for adherence across all users. Functional features and tracking are known to be useful for health users of mHealth apps [30,41]. The congruency of age or user group to purpose is once more reinforced as key in increased engagement, due to differences highlighted through app features. The results within age groups show that younger users were focused more on physical activity, as automatic activity plan generation was the most important feature for 18–24-year-old users and the least important for those between 55 and 64 years old. In the UK, where the shopping list feature was piloted, users found it to be helpful in meal adherence. Finally, people with poor-quality diets found tracking their daily stats and achievements more helpful than other user groups.

Our findings suggest that the future development of mHealth applications should be tailored to accommodate the specific needs of people in different gender, age, and user groups. More specifically, physical activity-related applications and features may be more relevant for male or younger users, as our study demonstrated higher adherence to recommendations for these groups than in women or older age groups. mHealth applications focusing on older age groups or on people living with a health condition should be designed to clearly support the designated conditions, as health management was found to be the priority of these groups. Furthermore, any mHealth application should facilitate engagement through clear goal-to-purpose in-app features, focusing on self-monitoring and tracking to support user engagement and facilitate successful goal regulation.

The limitations of this research include the execution and data collection taking place during the COVID-19 pandemic; the significantly reduced recruitment period of five months, due to pandemic restrictions, greatly impacted the project’s recruitment capabilities. Additionally, as mentioned in the methodology, the PROTEIN questionnaire and PROTEIN database were disconnected by design for anonymisation, data, and privacy protection purposes, and therefore specific user behaviour data could not be tied to user evaluation through the end-user questionnaire, which could lead to even better conclusions on the user level. Finally, since the focus of this research was to demonstrate the feasibility of the PROTEIN app in providing personalised nutrition and physical activity based on user acceptability and engagement, this study did not evaluate any medical history or nutritional or anthropometric parameter changes for any groups or investigate the impact of unnecessary or extreme goal setting on disordered eating, overexercising, or other unhealthy conditions.

Despite these limitations, our study is, to the best of our knowledge, one of the first to gather and analyse large-scale data through a personalised mHealth environment across different user groups in multiple EU countries. Furthermore, the results were drawn through both implicit and explicit means as we leveraged both self-reporting and a direct-use data end questionnaire, respectively.

## 5. Conclusions

Our study highlighted that, even within a personalised mHealth app environment, user engagement, behaviours, and meal adherence differ. Weight loss and health-related user goals and motivations were key drivers of engagement among all users of the PROTEIN app, with adherence further varying across different user demographics and age groups. Congruency between user groups and goals was key in demonstrating increased adherence, re-emphasising goal setting as a key factor in mHealth effectiveness. Furthermore, personalisation targeted at individual conditions is critical for behaviour change and intervention effectiveness in mHealth tools and/or applications. The research outcomes of this work can be used to inform future technological development of mHealth apps and enable researchers to better address personalisation, focusing on motivations and the specific needs of user groups. Additionally, the PROTEIN app and broader ecosystem established a framework for future research, leveraging AI in personalised nutrition and physical activity digital interventions.

## Figures and Tables

**Table 1:** Users’ demographics.

Variable	PROTEIN Database	PROTEIN End-User Questionnaire
	*n* (%)	*n* (%)
Total	579	446
Gender		
Male	255 (44.0)	163 (36.5)
Female	324 (56.0)	282 (63.2)
Rather not say	*-*	1 (0.2)
Age (years)		
18–24	90 (15.5)	47 (10.5)
25–34	157 (27.1)	129 (28.9)
35–44	150 (25.9)	134 (30.0)
45–54	76 (13.1)	74 (16.6)
55–64	76 (13.1)	37 (8.3)
>65	30 (5.2)	25 (5.6)
User Group		
Group A: Users with no health conditions, normal BMI, non-exercisers	289 (49.9)	129 (28.9)
Group B: Athletes and leisure exercisers	98 (16.9)	52 (11.7)
Group B: People with overweight	77 (13.3)	125 (28.0)
Group C: People with obesity	46 (7.9)	21 (4.7)
Group C: People with CVD	14 (2.4)	9 (2.0)
Group C: People with T2D	21 (3.6)	9 (2.0)
Group C: People with iron-deficiency anaemia	10 (1.7)	13 (2.9)
Group C: People with PQD	24 (4.1)	83 (18.6)
Other	N/A	5 (1.1)

CVD, cardiovascular disease; T2D, type 2 diabetes; PQD, poor-quality diet.

## Data Availability

The PROTEIN NAP database is publicly available at https://zenodo.org/records/7308053, accessed on 26 September 2024.

## Supplementary Materials

The following supporting information can be downloaded at: https://www.mdpi.com/article/10.3390/life14101238/s1, Table S1: Personal goals (AI advisor); Table S2: Self-reported goals (PROTEIN end-user questionnaire); Table S3: Inclusion and exclusion criteria—all pilots; Table S4: Significant differences by cell for personal user goals by gender; Table S5: Significant differences by cell for personal user goals by age group; Table S6: Significant differences by cell for personal user goals by user group; Table S7: Overall meal activity by goal; Table S8: Meal confirmations: pairwise comparisons for goals and gender; Table S9: Meal confirmations: pairwise comparisons for goals and age group; Table S10: Meal confirmations by user group.
