# Supplementary material for: Personal Goals, User Engagement, and Meal Adherence within a Personalised AI-Based Mobile Application for Nutrition and Physical Activity
Source: Life (Basel). 2024 Sep 27;14(10):1238. doi: 10.3390/life14101238 (PMC11508961; doi:10.3390/life14101238)
Supplement: Supplementary file 1 [file life-14-01238-s001.zip › H2020 PROTEIN Survey_17022022.pdf]

PROTEIN H2020
Questionnaire

Welcome!

The PROTEIN (PeRsOnalized nuTrition for hEalthy livINg", https://protein-h2020.eu/) project has received funding from the European Union's Horizon 2020 Research and Innovation Programme, under grant agreement No 817732.

This is an anonymous survey. No personal information will be collected. You will not be requested to disclose any personal information. The researchers will not be able to identify an individual respondent. IP logging is disabled. Data collected from the online survey will be kept for specific duration of the research period and will only be used for the purposes of the PROTEIN project. After that period, the data will be destroyed in line with the project's contractual obligations to the European Commission. Access to the data is exclusively allowed to authorized members of the PROTEIN Consortium, engaged in the aforementioned research activities. Participating in the following online survey is voluntary.

Please read the DISCLAIMER that follows and if you consent you can proceed with the questionnaire.

Thank you for considering to participate! Your feedback is important!

PERSONALISED NUTRITION FOR HEALTHY LIVING

PROTEIN H2020
Questionnaire

DISCLAIMER

* 1. DISCLAIMER: The PROTEIN (PeRsOnalized nuTrition for hEalthy livINg", https://protein-h2020.eu/) project has received funding from the European Union's Horizon 2020 Research and Innovation Programme, under grant agreement No 817732. PROTEIN is a research and innovation project that aims to promote an healthy lifestyle and help to improve the general state of health of the European population by combining the latest technologies to offer personalized nutrition and physical activity plans. The PROTEIN project runs from December 2018 to November 2022. Participating in the following online survey is purely voluntary, based on your informed consent. Your responses to the survey will be aggregated with the responses of other users, in order to produce statistical and significant information. The survey consists of 18 sections and will take around 15-20 minutes to be fill in, including free text and multiple choices questions. Please fill in the survey anonymously. You will not be requested to disclose any personal information. The researchers will not be able to identify an individual respondent. Thus, when drafting your responses, please abstain from including any information related to you or any other identified or identifiable person (name, email, phone number, etc). Data collected from the online survey will be kept for specific duration of the research period and will only be used for the aforementioned purposes. After that period, the data will be destroyed in line with the project's contractual obligations to the European Commission. Access to the data is exclusively allowed to authorized members of the PROTEIN Consortium, engaged in the aforementioned research activities. For more detailed information regarding the PROTEIN project, its Consortium and relevant activities, you can visit the project's website, communication channels, and social media (https://protein-h2020.eu/). Questions related to the processing of personal data can be directed to Eugenio Montavani at Eugenio.Mantovani@vub.be. For more information regarding the survey and the PROTEIN project please contact Lazaros Gymnopoulos at lazg@iti.gr. If you consider that your rights under the General Data Protection Regulation have been infringed as a result of the processing of personal data, you are also entitled to submit a complaint with a National Supervisory Authority. By clicking below "Yes! I consent" with the

Terms, you freely consent to take part in this survey and you confirm that you have read and fully understand the above statement.

- ◯ Yes! I consent. Let's go
- ◯ No! I do not consent

PROTEIN

PERSONALISED NUTRITION FOR HEALTHY LIVING

PROTEIN H2020
Questionnaire

General Information

Demographics

* 2. Please indicate your Age (in years):

- ◯ 18-24
- ◯ 25-34
- ◯ 35-44
- ◯ 45-54
- ◯ 55-64
- ◯ 65 and over

* 3. Please indicate your Gender:

- ◯ Male
- ◯ Female
- ◯ Rather not say

* 4. Please indicate your Education level:

- ◯ Less than High School
- ◯ High School
- ◯ Diploma
- ◯ Bachelor's degree
- ◯ Master's degree
- ◯ Doctoral degree, PhD

* 5. Please select your main Household income currency

- ◯ EUR-Euro
- ◯ GPD-British Pound
- ◯ USD-US Dollar
- ◯ AUD-Australlian Dollar
- ◯ Other (please specify)

* 6. Please indicate your Household income (in 2021):

- ◯ Less than 30,000
- ◯ 30,000-39,999
- ◯ 40,000-49,999
- ◯ 50,000-59,999
- ◯ 60,000-69,999
- ◯ 70,000 or more
- ◯ Rather not say

* 7. Please indicate your Marital status:

- ◯ Single
- ◯ Married
- ◯ Separated/Widowed
- ◯ Other (please specify)

* 8. Please indicate your Weight (in kgs):
[to convert pounds (lbs) to kilograms (kgr) go to https://www.unitconverters.net/weight-and-mass/lbs-to-kg.html]

* 9. Please indicate your Height (in cm):
[to convert feet (ft) and inches (in) to centimeters (cm) go to https://www.rapidtables.com/convert/length/feet-inch-to-cm.html]

* 10. Select the statement(s) that might apply:

- ☐ You are an Athlete (at a professional or semi-professional level)
- ☐ You are overweight (30kg/m2 > Body Mass Index (BMI) > 25 kg/m2)
- ☐ You have been diagnosed (by a professional) with Obesity (BMI > 30 kg/m2)
- ☐ You have been diagnosed (by a professional) with Diabetes
- ☐ You have been diagnosed (by a professional) with Cardiovascular disease
- ☐ You have been diagnosed (by a professional) with iron deficiency anaemia
- ☐ You consume less than 2-3 portions of fruit and vegetables a day
- ☐ Other (please specify)
- ☐ None of the above

* 11. Please indicate the organization that asked you to complete this survey:

- ◯ OCADO, United Kingdom
- ◯ University of Surrey (UoS), United Kingdom
- ◯ Katholieke Universiteit Leuven (KUL), Belgium
- ◯ Polo Europeo della Conoscenza (Europole), Italy
- ◯ European Association for the Study of Obesity (EASO), Ireland
- ◯ International Hellenic University (IHU), Greece
- ◯ AgriFood Capital (AFC), Netherlands
- ◯ Sport Lisboa e Benfica (SLB), Portugal
- ◯ Cognicase Management Consulting (CMC), Spain
- ◯ Centre for Research and Technology (CERTH), Greece
- ◯ Fluviale (FLUV), Italy
- ◯ BioSense Institute (BIOS), Serbia
- ◯ Universitätsmedizin Berlin (CHARITE), Germany
- ◯ Faculdade de Motricidade Humana, Universidade de Lisboa (FMH-UL), Portugal
- ◯ Other (please specify)

PROTEIN

PERSONALISED NUTRITION FOR HEALTHY LIVING

PROTEIN H2020
Questionnaire

Goals & Eating beliefs

Please indicate your goals and healthy eating beliefs related to the PROTEIN App use

* 12. What are your current health and well-being goals? (select all that apply)

- [ ] Eat more healthily
- [ ] Lose weight
- [ ] Be more active
- [ ] Manage an existing health condition
- [ ] Manage specific dietary requirements
- [ ] Improve energy levels
- [ ] Optimise/Improve my sports training
- [ ] Discover new healthy meal ideas
- [ ] Other (please specify)

* 13. Think about the PROTEIN App that you have used in the last 3 months and please rate the following statements.
Using the PROTEIN App has:

	Strongly Disagree	Somewhat Disagree	Neutral	Somewhat Agree	Strongly Agree
Increased my belief that poor diet/nutrition leads to disease	◯	◯	◯	◯	◯

	Strongly Disagree	Somewhat Disagree	Neutral	Somewhat Agree	Strongly Agree
Increased my belief that eating a healthy diet can prevent disease	◯	◯	◯	◯	◯
Increased my belief that diseases related to poor diet/nutrition are harmful	◯	◯	◯	◯	◯
Increase my belief that eating a healthy diet is important in preventing disease	◯	◯	◯	◯	◯
Increased my motivation to eat a healthy diet	◯	◯	◯	◯	◯
Increased my ability to eat a healthy diet	◯	◯	◯	◯	◯
Increased my confidence that I can eat a healthy diet	◯	◯	◯	◯	◯
Increased my desire to eat a healthy diet	◯	◯	◯	◯	◯
Increased my intentions to eat a healthy diet	◯	◯	◯	◯	◯
Increased my attitudes about the importance of eating a healthy diet in preventing disease	◯	◯	◯	◯	◯

	Strongly Disagree	Somewhat Disagree	Neutral	Somewhat Agree	Strongly Agree
Increased my belief that people important to me want me to eat a healthy diet	◯	◯	◯	◯	◯
Increased my perception that many other people are eating a healthy diet	◯	◯	◯	◯	◯
Increased my knowledge of the diseases that are caused by poor diet/nutrition	◯	◯	◯	◯	◯
Increased my knowledge of the ways in which I can eat a healthy diet	◯	◯	◯	◯	◯
Increased my awareness of the benefits of eating a healthy diet Increased my desire to be healthy	◯	◯	◯	◯	◯
Increased the social support I have received for eating a healthy diet	◯	◯	◯	◯	◯
Increased the positive feedback I have received for eating a healthy diet	◯	◯	◯	◯	◯

	Strongly Disagree	Somewhat Disagree	Neutral	Somewhat Agree	Strongly Agree
Increased my desire to set goals to eat a healthy diet Increased my ability to achieve my healthy diet goals	○	○	○	○	○
Increased my awareness of my eating intentions/meal plans	○	○	○	○	○
Increased my belief that awareness of my actual eating behavior helps me to follow a healthily diet	○	○	○	○	○

*** 14. Think about the PROTEIN App that you have used in the last 3 months and please rate the following statements.**
Using the PROTEIN App has:

	Strongly Disagree	Somewhat Disagree	Neutral	Somewhat Agree	Strongly Agree
Increased my actual goal setting to eat a healthy diet	◯	◯	◯	◯	◯
Increased my frequency of eating healthy foods	◯	◯	◯	◯	◯
Increased my consistency in eating healthy foods	◯	◯	◯	◯	◯
Increased my resistance to tempting food	◯	◯	◯	◯	◯
Led to changes to achieve agreement between what I am actually eating and what I intended/planned to eat	◯	◯	◯	◯	◯

PROTEIN
PERSONALISED NUTRITION FOR HEALTHY LIVING

PROTEIN H2020 Questionnaire

Physical activity beliefs

Please indicate your Physical activity beliefs related to the PROTEIN App use

* 15. Think about the PROTEIN App that you have used in the last 3 months and please rate the following statements.
Using the PROTEIN App has:

	Strongly Disagree	Somewhat Disagree	Neutral	Somewhat Agree	Strongly Agree
Increased my belief that sedentary lifestyle leads to disease	◯	◯	◯	◯	◯
Increased my belief that being physically active can prevent disease	◯	◯	◯	◯	◯
Increased my belief that diseases related to sedentary lifestyle are harmful	◯	◯	◯	◯	◯
Increase my belief that being physically active is important in preventing disease	◯	◯	◯	◯	◯

	Strongly Disagree	Somewhat Disagree	Neutral	Somewhat Agree	Strongly Agree
Increased my motivation to be physically active	◯	◯	◯	◯	◯
Increased my ability to be physically active	◯	◯	◯	◯	◯
Increased my confidence that I can be physically active	◯	◯	◯	◯	◯
Increased my desire to be physically active	◯	◯	◯	◯	◯
Increased my intentions to be physically active	◯	◯	◯	◯	◯
Increased my attitudes about the importance of being physically active in preventing disease	◯	◯	◯	◯	◯
Increased my belief that people important to me want me to be physically active	◯	◯	◯	◯	◯
Increased my perception that many other people are being physically active	◯	◯	◯	◯	◯

	Strongly Disagree	Somewhat Disagree	Neutral	Somewhat Agree	Strongly Agree
Increased my knowledge of the diseases that are caused be sedentary lifestyle	◯	◯	◯	◯	◯
Increased my knowledge of the ways in which I can be physically active	◯	◯	◯	◯	◯
Increased my awareness of the benefits of being physically active	◯	◯	◯	◯	◯
Increased my desire to be physically healthy	◯	◯	◯	◯	◯
Increased the social support I have received for being physically active	◯	◯	◯	◯	◯
Increased the positive feedback I have received for being physically active	◯	◯	◯	◯	◯
Increased my desire to set goals to be physically active	◯	◯	◯	◯	◯
Increased my ability to achieve my physical activity goals	◯	◯	◯	◯	◯

	Strongly Disagree	Somewhat Disagree	Neutral	Somewhat Agree	Strongly Agree
Increased my awareness of my physical activity intentions/plans	◯	◯	◯	◯	◯
Increased my belief that awareness of my actual physical activity helps me to be more physically active	◯	◯	◯	◯	◯

*** 16. Think about the PROTEIN App that you have used in the last 3 months and please rate the following statements.**
Using the PROTEIN App has:

	Strongly Disagree	Somewhat Disagree	Neutral	Somewhat Agree	Strongly Agree
Increased my actual goal setting to be physically active	◯	◯	◯	◯	◯
Increased my frequency of being physically active	◯	◯	◯	◯	◯
Increased my consistency in being physically active	◯	◯	◯	◯	◯
Increased my resistance of being sedentary	◯	◯	◯	◯	◯
Led to changes to achieve agreement between my actual physical activity levels and what I intended/planned to do	◯	◯	◯	◯	◯

PROTEIN H2020 Questionnaire

Hydration beliefs

Please indicate your Hydration beliefs related to the PROTEIN App use

* 17. Think about the PROTEIN App that you have used in the last 3 months and please rate the following statements.
Using the PROTEIN App has:

	Strongly Disagree	Somewhat Disagree	Neutral	Somewhat Agree	Strongly Agree
Increased my belief that inadequate hydration leads to disease Increased my belief that having a healthy level of hydration can prevent disease	◯	◯	◯	◯	◯
Increased my belief that diseases related to inadequate hydration are	◯	◯	◯	◯	◯
harmful	◯	◯	◯	◯	◯
Increase my belief that having a healthy hydration is important in	◯	◯	◯	◯	◯
preventing disease	◯	◯	◯	◯	◯

	Strongly Disagree	Somewhat Disagree	Neutral	Somewhat Agree	Strongly Agree
Increased my motivation to have a healthy level of hydration	◯	◯	◯	◯	◯
Increased my ability to have a healthy level of hydration	◯	◯	◯	◯	◯
Increased my confidence that I can have a healthy level of hydration	◯	◯	◯	◯	◯
Increased my desire to have a healthy level of hydration	◯	◯	◯	◯	◯
Increased my intentions to have a healthy level of hydration	◯	◯	◯	◯	◯
Increased my attitudes about the importance of having a healthy level of hydration in preventing disease	◯	◯	◯	◯	◯
Increased my belief that people important to me want me to have a healthy level of hydration	◯	◯	◯	◯	◯
Increased my perception that many other people are having a healthy level of hydration	◯	◯	◯	◯	◯

	Strongly Disagree	Somewhat Disagree	Neutral	Somewhat Agree	Strongly Agree
Increased my knowledge of the diseases that are caused by inadequate hydration	○	○	○	○	○
Increased my knowledge of the ways in which I can have a healthy level of hydration	○	○	○	○	○
Increased my awareness of the benefits of having a healthy level of hydration	○	○	○	○	○
Increased my desire to be well hydrated	○	○	○	○	○
Increased the social support I have received for having a healthy level of hydration	○	○	○	○	○
Increased the positive feedback I have received for having a healthy level of hydration	○	○	○	○	○
Increased my desire to set goals to have a healthy level of hydration	○	○	○	○	○
Increased my ability to achieve my healthy hydration goals	○	○	○	○	○
Increased my awareness of my intended/planned hydration levels	○	○	○	○	○

	Strongly Disagree	Somewhat Disagree	Neutral	Somewhat Agree	Strongly Agree
Increased my belief that awareness of my actual hydration level helps me to have a healthy level of hydration	◯	◯	◯	◯	◯

*** 18. Think about the PROTEIN App that you have used in the last 3 months and please rate the following statements.**
Using the PROTEIN App has:

	Strongly Disagree	Somewhat Disagree	Neutral	Somewhat Agree	Strongly Agree
Increased my actual goal setting to have a healthy level of hydration	◯	◯	◯	◯	◯
Increased my frequency of having healthy hydration	◯	◯	◯	◯	◯
Increased my consistency in having healthy hydration	◯	◯	◯	◯	◯
Increased my resistance to poor hydration levels	◯	◯	◯	◯	◯
Led to changes to achieve agreement between my actual hydration level and the one intended/planned to	◯	◯	◯	◯	◯

PERSONALISED NUTRITION FOR HEALTHY LIVING

PROTEIN H2020 Questionnaire

Acceptance of the PROTEIN App

Here we aim to understand your acceptance of the PROTEIN App

*** 19. Think about the PROTEIN App that you have used in the last 3 months and please rate the following statements:**

	Strongly Disagree	Somewhat Disagree	Neutral	Somewhat Agree	Strongly Agree
The PROTEIN App was useful	○	○	○	○	○
The PROTEIN App was easy to use	○	○	○	○	○
I enjoyed using the PROTEIN App	○	○	○	○	○
I am satisfied with my use of the PROTEIN App	○	○	○	○	○
I liked the PROTEIN App	○	○	○	○	○
I would recommend the PROTEIN App to others	○	○	○	○	○

PROTEIN

PERSONALISED NUTRITION FOR HEALTHY LIVING

PROTEIN H2020
Questionnaire

Usefulness of Meal Plans

Here we aim to understand how useful is the information related to the meal plans provided by the PROTEIN App

* 20. Please rate the following statements regarding the characteristics of the PROTEIN App meal plans:

	Strongly Disagree	Somewhat Disagree	Neutral	Somewhat Agree	Strongly Agree
Meal suggestions reflected my habits and culture	○	○	○	○	○
Meal suggestions were easy to prepare	○	○	○	○	○
Meal suggestions were convenient	○	○	○	○	○
Meals and ingredients were affordable to prepare and procure	○	○	○	○	○
Meals and ingredients were in accordance with my dietary preferences	○	○	○	○	○
Meals were satiating enough	○	○	○	○	○
Meals and ingredients were available to find where I live	○	○	○	○	○
Portion sizes fitted in with my preferences	○	○	○	○	○
Meal plans supported an healthy lifestyle	○	○	○	○	○

* 21. Do you feel the PROTEIN App offers sufficient variety in your meal plan?

No variety at all — Great variety

☆ ☆ ☆ ☆ ☆

* 22. Which of the following helped you to follow the meal plan? (select all that apply)

- [ ] New suggestions for healthy meals
- [ ] Ease of tracking calorie intake
- [ ] Meal plans automatically generated from the user profile
- [ ] Activity plans automatically generated from the user profile
- [ ] Shopping lists generated from meal plans
- [ ] Ease of tracking water consumption
- [ ] The dining-out feature
- [ ] Nutrition information on meals
- [ ] Daily stats and achievements
- [ ] Other (please specify)

* 23. How did you find the option of recording meals within the PROTEIN app?

Very difficult — Very easy — N/A

☆ ☆ ☆ ☆ ☆ ○

* 24. How often did you follow the recommended meal plans?

- ○ Never
- ○ Rarely
- ○ Sometimes
- ○ Usually
- ○ Always

PROTEIN

PERSONALISED NUTRITION FOR HEALTHY LIVING

PROTEIN H2020
Questionnaire

Usefulness of Meal Plans

25. What would help you to follow the recommended meal plans more?

PERSONALISED NUTRITION FOR HEALTHY LIVING

PROTEIN H2020 Questionnaire

Usefulness of Activity Plans

Here we aim to understand how useful the information related to the physical activity plans provided by the PROTEIN App is

* 26. Please rate the following statements regarding the characteristics of the PROTEIN App physical activity plans:

	Strongly Disagree	Somewhat Disagree	Neutral	Somewhat Agree	Strongly Agree
Physical activity suggestions were familiar to my habits and culture	○	○	○	○	○
Physical activity suggestions were easy to perform	○	○	○	○	○
Physical activity suggestions were convenient	○	○	○	○	○
Physical activity plans fitted my preferences	○	○	○	○	○
Physical activity plans supported an active lifestyle	○	○	○	○	○

* 27. How did you find recording physical activity within the PROTEIN app?

Very difficult ☆ ☆ ☆ ☆ ☆ Very easy

* 28. How often did you follow the recommended physical activity plans?

- ◯ Never
- ◯ Rarely
- ◯ Sometimes
- ◯ Usually
- ◯ Always

PERSONALISED NUTRITION FOR HEALTHY LIVING

PROTEIN H2020
Questionnaire

Usefulness of Activity Plans

29. What would help you to follow the recommended physical activity plans more?

PERSONALISED NUTRITION FOR HEALTHY LIVING

PROTEIN H2020 Questionnaire

PROTEIN App Usability

Here we aim to understand the attributes of PROTEIN App usability

* 30. Do you agree with the statement: The PROTEIN App is sufficiently personalised to your individual requirements and routines?

Strongly Disagree	Somewhat Disagree	Neutral	Somewhat Agree	Strongly Agree
○	○	○	○	○

* 31. Which new aspects of the PROTEIN App could help you to adopt/take steps towards a healthier lifestyle? (select all that apply)

- ☐ Additional video, text and images for suggested physical activity
- ☐ Ability to select my preferred exercises (e.g., running, swimming, cycling)
- ☐ More meals from my country or region
- ☐ Larger variety of meals
- ☐ Ability to select weekly workout routines
- ☐ Ability to track my weight
- ☐ Reminders to confirm meals eaten
- ☐ Other (please specify)

* 32. Do you agree that the PROTEIN App could help you in the future in maintaining a healthy lifestyle?

Strongly Disagree	Somewhat Disagree	Neutral	Somewhat Agree	Strongly Agree
○	○	○	○	○

*** 33. Did you encounter any unexpected behaviour (e.g., exits) while using the PROTEIN App?**

- ◯ Never
- ◯ Yes

PROTEIN

PERSONALISED NUTRITION FOR HEALTHY LIVING

PROTEIN H2020
Questionnaire

Unexpected behaviour frequency

* 34. Please indicate the frequency of encountered errors:

Always				Rarely
☆	☆	☆	☆	☆

* 35. Think about the PROTEIN App that you have used in the last 3 months and please rate the following statements:

	Strongly Disagree	Somewhat Disagree	Neutral	Somewhat Agree	Strongly Agree
I lost myself in this experience	◯	◯	◯	◯	◯
The time I spent using the PROTEIN App just slipped away	◯	◯	◯	◯	◯
I was absorbed in this experience	◯	◯	◯	◯	◯
I felt frustrated while using the PROTEIN App	◯	◯	◯	◯	◯
I found the PROTEIN App confusing to use	◯	◯	◯	◯	◯
Using the PROTEIN App was taxing	◯	◯	◯	◯	◯
The PROTEIN App was attractive	◯	◯	◯	◯	◯
The PROTEIN App was aesthetically appealing	◯	◯	◯	◯	◯
The PROTEIN App appealed to my senses	◯	◯	◯	◯	◯
Using the PROTEIN App was worthwhile	◯	◯	◯	◯	◯
My experience was rewarding	◯	◯	◯	◯	◯
I felt interested in this experience	◯	◯	◯	◯	◯

PROTEIN

PERSONALISED NUTRITION FOR HEALTHY LIVING

PROTEIN H2020
Questionnaire

Feedback

Any additional feedback

36. Please provide any additional feedback on what you enjoyed MOST/LEAST about using the PROTEIN App. Any other comments, improvements, bug reports or issues related with this App would be most welcome!

PROTEIN

PERSONALISED NUTRITION FOR HEALTHY LIVING

PROTEIN H2020 Questionnaire

PROTEIN App dissemination (1)

Here we aim to understand the marketability and dissemination of the PROTEIN App

* 37. Are you aware of other apps that offer similar features (e.g., meal adaptation) compared to the PROTEIN App?

- ◯ No, PROTEIN App is unique
- ◯ A few only
- ◯ Several exist
- ◯ N/A: I don't know

PROTEIN

PERSONALISED NUTRITION FOR HEALTHY LIVING

PROTEIN H2020 Questionnaire

Other mobile apps

38. If you are aware of other mobile apps that are currently available, please specify below:

PROTEIN

PERSONALISED NUTRITION FOR HEALTHY LIVING

PROTEIN H2020 Questionnaire

PROTEIN App dissemination (2)

Here we aim to understand the marketability and dissemination of the PROTEIN App

* 39. Please rate below your willingness to pay for the PROTEIN App (if commercialised after the end of the project).

Not at all				Definitely
★	★	★	★	★

* 40. Would any of the following encourage you to pay for the PROTEIN App (either as a one-off purchase or as subscription payment)?
(select all that apply)

- [ ] Satisfied from a 30-day trial period
- [ ] A larger selection of healthy meal recipes
- [ ] A wider selection of tailored physical activity plans
- [ ] Discounts or special promotions on healthy meal purchases through using the app
- [ ] Availability of shopping purchases from ANY of the main online grocery retailers
- [ ] Get tailored plans from healthcare experts through the app
- [ ] Recommended by a doctor or healthcare provider
- [ ] Ability to interact with other users
- [ ] Other (please specify)

PROTEIN

PERSONALISED NUTRITION FOR HEALTHY LIVING

PROTEIN H2020
Questionnaire

Thank you!

Thank you for taking part! Click the "submit" button below to exit.
