# Supplementary material for: Personal Goals, User Engagement, and Meal Adherence within a Personalised AI-Based Mobile Application for Nutrition and Physical Activity
Source: Life (Basel). 2024 Sep 27;14(10):1238. doi: 10.3390/life14101238 (PMC11508961; doi:10.3390/life14101238)
Supplement: Supplementary file 1 [file life-14-01238-s001.zip › Supplementary Material File.pdf]

A – Personal Goals

Table S1. Personal Goals (AI Advisor)

a/a	Goal	Type
**1**	Increase fiber intake	Health
**2**	Increase folic acid intake	Health
**3**	Increase fruit intake	Health
**4**	Increase fluid intake	Health
**5**	Increase vegetable intake	Health
**6**	Increase vitamin A intake	Health
**7**	Increase vitamin B12 intake	Health
**8**	Increase vitamin C intake	Health
**9**	Increase vitamin D intake	Health
**10**	Increase Iron intake	Health
**11**	Decrease Sodium intake	Health
**12**	Increase fat intake	Health
**13**	Increase electrolytes intake	Health
**14**	Increase energy expenditure	Physical Activity
**15**	Increase activity duration	Physical Activity
**16**	Increase activity intensity	Physical Activity
**17**	Increase body weight	Weight
**18**	Maintain body weight	Weight
**19**	Increase body fat	Weight

**20**	Decrease body weight	Weight
**21**	Decrease energy expenditure	Weight
**22**	Decrease body fat	Weight
**23**	Decrease fat intake	Weight
**24**	Increase muscle mass	Health, Weight
**25**	Decrease carbohydrates intake	Health, Weight
**26**	Increase protein intake	Health, Weight

Table S2. Self-reported goals (PROTEIN End user questionnaire)

**a/a**	**Goal**	**Type**
**1**	Eat more healthily	Health
**2**	Manage an existing health condition	Health
**3**	Manage specific dietary requirements	Health
**4**	Lose weight	Weight Loss
**5**	Be more active	Physical Activity
**6**	Improve energy levels	Physical Activity
**7**	Optimise/Improve my sports training	Physical Activity
**8**	Discover new healthy meal ideas	Nutrition
**9**	Other	-

B – Pilot Criteria

Table S3. Inclusion and exclusion criteria – All pilots

Group	Criteria	
	Inclusion	Exclusion
**Group A - Users with no health conditions, normal BMI and non-exercisers**	• BMI < 30kg/m2<br>• Existing Ocado customers (UK online food store pilot)	• Chronic diseases or health conditions associated with pilots in Group C (e.g. People with T2D, CVD, PQD)<br>• Food intolerances<br>• Allergies
**Group B**		
**Athletes or leisure exercisers**	**Athletes**<br>• Active athletic performance or member of an athletic team<br><br>**Leisure exercisers**<br>• > 3 hours regular exercise/week)	**Athletes**<br>• No major health conditions or currently receiving treatment for medical conditions<br><br>**Leisure exercisers**<br>• Those with major health conditions or currently receiving treatment for medical conditions

**Individuals with overweight**	• 25 ≤ BMI < 30 kg/m2<br>• No major health conditions or currently receiving treatment for medical conditions	• Those who have been diagnosed with CVD, T1D or T2D<br>• Clinically significant hematological abnormalities or an active malignancy<br>• Regular exercisers/ people who are unable to exercise for medical purposes or otherwise indicated by a health professional
**Group C**		
**People with obesity**	• BMI ≥ 30 kg/m2<br>• Stable in terms of symptoms and treatment<br>• No major health conditions and not currently receiving treatment for medical conditions	• Those who have been diagnosed with CVD, T1D or T2D<br>• Clinically significant hematological abnormalities or an active malignancy

**People with CVD**	• CVD diagnosis<br>• Stable in terms of symptoms and treatment	• Clinically significant hematological abnormalities or an active malignancy
**People with type 2 diabetes**	• Prediabetes or T2D diagnosis	• Insulin dependent T1D or T2D<br>• Severe kidney and/or liver disease<br>• Existing therapy with steroids<br>• Malignant disease<br>• Severe CVD

**People with iron deficiency anaemia**	• Diagnosed iron deficiency<br>• In good physical health	• Those who have been diagnosed with CVD, T1D or T2D<br>• Major health conditions or currently receiving treatment for medical conditions<br>• Clinically significant hematological abnormalities (excluding iron deficiency anaemia) or an active malignancy<br>• Regular exercisers/ people who are unable to exercise for medical purposes or otherwise indicated by a health professional
**People with PQD (<3 portions of fruit/vegetables per day)**	• Low fruit and vegetable intakes<br>• In good physical health	• Those who have been diagnosed with CVD, T1D or T2D<br>• Major health conditions or currently receiving treatment for medical conditions<br>• Clinically significant hematological abnormalities (excluding iron deficiency anaemia) or an active malignancy<br>• Regular exercisers/ people who are unable to exercise for medical purposes or otherwise indicated by a health professional

CVD: Cardiovascular Diseases, T1D: Type 1 Diabetes, T2D: Type 2 Diabetes, PQD: Poor Quality Diets

C – Statistical Tests Outputs

Table S4. Significant differences by cell for personal user goals by gender

	Other (please specify)	Manage an existing health condition	Manage specific dietary requirements	Optimise/ Improve my sports training	Discover new healthy meal ideas	Improve energy levels	Lose weight	Be more active	Eat more healthily
Male	>	<	>	>	<	<	<	<	<
Female	<	>	<	<	>	>	>	>	>
Rather not say	<	<	<	<	<	>	>	>	>

	Other (please specify)	Manage an existing health condition	Manage specific dietary requirements	Optimise/ Improve my sports training	Discover new healthy meal ideas	Improve energy levels	Lose weight	Be more active	Eat more healthily
Male	0,012	0,890	0,061	0,071	0,802	0,409	0,519	0,572	0,789
Female	0,012	0,890	0,062	0,088	0,739	0,410	0,520	0,621	0,789
Rather not say	1,000	1,000	1,000	1,000	1,000	0,488	0,533	0,546	1,000

Note: Fisher's exact test output: Significance by cell indicates if the actual value is equal (=), lower (<) or higher (>) than the theoretical value; Values displayed in red are significant: $p \leq 0.05$; Grey highlight: actual value higher (>) than theoretical

Table S5. Significant differences by cell for personal user goals by age group

	Other	Manage an existing health condition	Manage specific dietary requirements	Optimise/Improve my sports training	Discover new healthy meal ideas	Improve energy levels	Lose weight	Be more active	Eat more healthily
18-24	>	<	<	>	<	<	<	>	>
25-34	>	<	>	>	>	<	<	<	<
35-44	<	>	<	<	>	>	<	>	<
45-54	>	<	<	>	<	>	<	<	>
55-64	>	>	<	<	<	<	>	<	<
> 65	>	>	>	<	<	<	>	<	>

	Other	Manage an existing health condition	Manage specific dietary requirements	Optimise/Improve my sports training	Discover new healthy meal ideas	Improve energy levels	Lose weight	Be more active	Eat more healthily
18-24	0,596	0,650	1,000	0,008	0,220	0,393	0,816	0,565	0,913
25-34	1,000	0,472	0,676	0,602	0,339	0,938	0,455	0,941	1,000
35-44	0,287	0,670	0,586	0,099	0,197	0,758	0,605	0,382	0,890
45-54	0,667	0,729	1,000	0,803	0,349	0,190	1,000	0,724	0,867
55-64	0,467	0,591	0,797	0,338	0,633	0,775	0,027	0,686	1,000
65 and ove	0,324	0,035	0,189	0,813	0,692	0,474	0,495	0,317	1,000

Note: Fisher's exact test output: Significance by cell indicates if the actual value is equal (=), lower (<) or higher (>) than the theoretical value; Values displayed in red are significant: p ≤ 0.05; Grey highlight: actual value higher (>) than theoretical

Table S6. Significant differences by cell for personal user goals by user group

	Eat more healthily	Lose weight	Be more active	Manage an existing health condition	Manage specific dietary requirements	Improve energy levels	Optimise/ Improve my sports training	Discover new healthy meal ideas	Other
People with obesity	<	>	<	>	>	<	<	<	<
Individuals with overweight	<	>	<	<	<	>	<	<	<
Athletes & exercisers	<	<	>	<	>	<	>	<	<
People with poor quality diets	>	<	>	<	<	<	<	>	<
People with iron deficiency anaemia	<	>	>	>	>	>	<	<	<
People with cardiovascular disease	<	>	<	>	>	<	<	<	<
People with type 2 diabetes	<	<	<	>	>	<	>	>	<
General public	>	<	>	<	<	>	>	>	>

	Eat more healthily	Lose weight	Be more active	Manage an existing health condition	Manage specific dietary requirements	Improve energy levels	Optimise/ Improve my sports training	Discover new healthy meal ideas	Other
People with obesity	0,382	0,006	0,062	0,016	0,081	0,046	1,000	0,857	1,000
Individuals with overweight	1,000	0,000	0,627	0,308	0,454	1,000	0,053	0,377	1,000
Athletes & exercisers	1,000	0,008	0,503	1,000	0,231	0,481	<0,0001	0,368	0,613
People with poor quality diets	0,601	0,533	0,096	0,382	0,571	0,933	0,104	0,463	1,000
People with iron deficiency anaemia	0,590	1,000	0,849	0,275	0,493	0,542	1,000	0,508	1,000
People with cardiovascular disease	0,661	0,359	0,252	0,014	0,195	0,808	1,000	0,790	1,000
People with type 2 diabetes	0,784	1,000	0,037	0,012	0,014	0,351	0,661	0,312	1,000
General public	0,545	0,001	0,776	0,296	0,124	0,070	0,466	0,187	0,085

Note: Fisher's exact test output: Significance by cell indicates if the actual value is equal (=), lower (<) or higher (>) than the theoretical value; Values displayed in red are significant: p ≤ 0.05; Grey highlight: actual value higher (>) than theoretical

Table S7. Overall meal activity by goal

	Confirmed	Rejected
Increase energy expenditure	>	<
Increase fiber intake	>	<
Increase folic acid intake	>	<
Increase fruit intake	<	>
Increase fluid intake	>	<
Increase vegetable intake	<	>
Increase vitamin A intake	<	>
Increase vitamin B12 intake	<	>
Increase vitamin C intake	>	<
Increase vitamin D intake	<	>
Increase activity duration	>	<
Increase Iron intake	<	>
Increase activity intensity	>	<
Increase body weight	>	<
Maintain body weight	>	<
Increase body fat	>	<
Decrease body weight	<	>
Decrease energy expenditure	>	<
Decrease body fat	>	<
Decrease carbohydrates intake	>	<
Decrease fat intake	>	<
Increase muscle mass	<	>
Decrease protein intake	<	>
Decrease Sodium intake	>	<
Increase fat intake	>	<
Increase protein intake	>	<
Increase electrolytes intake	<	>

	Confirmed	Rejected
Increase energy expenditure	0,528	0,528
Increase fiber intake	0,122	0,122
Increase folic acid intake	0,867	0,867
Increase fruit intake	0,001	0,001
Increase fluid intake	0,731	0,731
Increase vegetable intake	<0,0001	<0,0001
Increase vitamin A intake	0,412	0,412
Increase vitamin B12 intake	<0,0001	<0,0001
Increase vitamin C intake	0,917	0,917
Increase vitamin D intake	0,008	0,008
Increase activity duration	0,696	0,696
Increase Iron intake	0,542	0,542
Increase activity intensity	0,000	0,000
Increase body weight	<0,0001	<0,0001
Maintain body weight	<0,0001	<0,0001
Increase body fat	0,878	0,878
Decrease body weight	<0,0001	<0,0001
Decrease energy expenditure	<0,0001	<0,0001
Decrease body fat	<0,0001	<0,0001
Decrease carbohydrates intake	<0,0001	<0,0001
Decrease fat intake	0,077	0,077
Increase muscle mass	0,013	0,013
Decrease protein intake	0,002	0,002
Decrease Sodium intake	0,002	0,002
Increase fat intake	0,552	0,552
Increase protein intake	0,091	0,091
Increase electrolytes intake	0,575	0,575

Note: Fisher's exact test output: Significance by cell indicates if the actual value is equal (=), lower (<) or higher (>) than the theoretical value; Values displayed in red are significant: $p \leq 0.05$; Grey highlight: actual value higher (>) than theoretical

Table S8. Meal confirmations: Pairwise comparisons for goals and gender

	MEN	WOMEN		MEN	WOMEN
Increase energy expenditure	>	<	Increase energy expenditure	<0,0001	<0,0001
Increase fiber intake	<	>	Increase fiber intake	<0,0001	<0,0001
Increase folic acid intake	<	>	Increase folic acid intake	<0,0001	<0,0001
Increase fruit intake	>	<	Increase fruit intake	<0,0001	<0,0001
Increase fluid intake	<	>	Increase fluid intake	<0,0001	<0,0001
Increase vegetable intake	<	>	Increase vegetable intake	0,611	0,611
Increase vitamin A intake	<	>	Increase vitamin A intake	<0,0001	<0,0001
Increase vitamin B12 intake	<	>	Increase vitamin B12 intake	<0,0001	<0,0001
Increase vitamin C intake	<	>	Increase vitamin C intake	0,013	0,013
Increase vitamin D intake	<	>	Increase vitamin D intake	0,001	0,001
Increase activity duration	>	<	Increase activity duration	<0,0001	<0,0001
Increase Iron intake	<	>	Increase Iron intake	<0,0001	<0,0001
Increase activity intensity	>	<	Increase activity intensity	<0,0001	<0,0001
Increase body weight	<	>	Increase body weight	0,051	0,051
Maintain body weight	>	<	Maintain body weight	<0,0001	<0,0001
Increase body fat	<	>	Increase body fat	<0,0001	<0,0001
Decrease body weight	<	>	Decrease body weight	<0,0001	<0,0001
Decrease energy expenditure	<	>	Decrease energy expenditure	0,016	0,016
Decrease body fat	>	<	Decrease body fat	<0,0001	<0,0001
Decrease carbohydrates intake	<	>	Decrease carbohydrates intake	0,000	0,000
Decrease fat intake	>	<	Decrease fat intake	0,183	0,183
Increase muscle mass	<	>	Increase muscle mass	<0,0001	<0,0001
Decrease Sodium intake	<	>	Decrease Sodium intake	<0,0001	<0,0001
Increase fat intake	<	>	Increase fat intake	<0,0001	<0,0001
Increase protein intake	<	>	Increase protein intake	0,153	0,153
Increase electrolytes intake	<	>	Increase electrolytes intake	<0,0001	<0,0001

Note: Fisher's exact test output: Significance by cell indicates if the actual value is equal (=), lower (<) or higher (>) than the theoretical value; Values displayed in red are significant: $p \leq 0.05$; Grey highlight: actual value higher (>) than theoretical

Table S9. Meal confirmations: Pairwise comparisons for goals age group

	18-24	25-34	35-44	45-54	55-64	>65		18-24	25-34	35-44	45-54	55-64	>65
Increase body fat	<	<	<	>	<	<	Increase body fat	<0,0001	<0,0001	<0,0001	<0,0001	<0,0001	0,016
Increase fat intake	<	<	<	>	<	<	Increase fat intake	<0,0001	<0,0001	<0,0001	<0,0001	<0,0001	0,011
Increase vitamin A intake	<	>	>	<	<	<	Increase vitamin A intake	<0,0001	0,196	<0,0001	0,085	<0,0001	0,001
Decrease Sodium intake	<	<	<	>	>	<	Decrease Sodium intake	<0,0001	<0,0001	<0,0001	<0,0001	<0,0001	0,001
Decrease energy expenditure	<	>	>	<	<	<	Decrease energy expenditure	<0,0001	<0,0001	0,227	0,274	<0,0001	<0,0001
Increase electrolytes intake	<	<	>	>	<	<	Increase electrolytes intake	<0,0001	<0,0001	0,732	<0,0001	<0,0001	<0,0001
Increase body weight	<	<	>	>	<	<	Increase body weight	0,000	<0,0001	<0,0001	0,012	<0,0001	<0,0001
Increase folic acid intake	<	<	>	>	<	<	Increase folic acid intake	<0,0001	<0,0001	<0,0001	<0,0001	<0,0001	<0,0001
Decrease fat intake	<	<	>	>	>	<	Decrease fat intake	<0,0001	<0,0001	<0,0001	0,001	<0,0001	<0,0001
Increase vitamin C intake	<	<	>	<	<	<	Increase vitamin C intake	<0,0001	<0,0001	<0,0001	0,034	<0,0001	<0,0001
Increase Iron intake	>	<	<	>	>	<	Increase Iron intake	<0,0001	<0,0001	0,415	0,079	0,330	0,002
Increase vitamin D intake	>	>	>	<	>	<	Increase vitamin D intake	<0,0001	<0,0001	0,141	<0,0001	0,520	<0,0001
Increase vitamin B12 intake	<	>	>	<	<	<	Increase vitamin B12 intake	<0,0001	0,000	<0,0001	<0,0001	0,791	<0,0001
Increase protein intake	<	>	<	>	<	<	Increase protein intake	<0,0001	0,033	<0,0001	<0,0001	<0,0001	<0,0001
Increase fiber intake	<	<	<	>	>	<	Increase fiber intake	<0,0001	<0,0001	0,011	0,121	<0,0001	<0,0001
Increase activity intensity	>	>	>	<	<	<	Increase activity intensity	0,018	<0,0001	0,445	0,182	<0,0001	<0,0001
Increase energy expenditure	<	>	<	>	<	<	Increase energy expenditure	<0,0001	<0,0001	0,057	<0,0001	<0,0001	<0,0001
Increase muscle mass	>	<	<	>	<	<	Increase muscle mass	<0,0001	0,529	<0,0001	<0,0001	<0,0001	<0,0001
Decrease carbohydrates intake	<	<	<	>	>	>	Decrease carbohydrates intake	<0,0001	<0,0001	<0,0001	<0,0001	<0,0001	<0,0001
Increase fruit intake	<	<	>	<	<	<	Increase fruit intake	<0,0001	<0,0001	<0,0001	0,710	0,001	0,021
Increase fluid intake	>	<	>	<	<	<	Increase fluid intake	<0,0001	<0,0001	<0,0001	0,002	<0,0001	<0,0001
Increase vegetable intake	>	<	<	<	>	<	Increase vegetable intake	<0,0001	<0,0001	0,248	<0,0001	<0,0001	0,001
Maintain body weight	>	>	<	<	<	>	Maintain body weight	<0,0001	<0,0001	<0,0001	<0,0001	<0,0001	<0,0001
Increase activity duration	>	>	<	<	<	<	Increase activity duration	<0,0001	<0,0001	0,120	<0,0001	<0,0001	<0,0001
Decrease body fat	<	>	>	<	>	>	Decrease body fat	<0,0001	<0,0001	0,000	<0,0001	<0,0001	<0,0001
Decrease body weight	>	<	<	<	>	>	Decrease body weight	<0,0001	<0,0001	<0,0001	<0,0001	<0,0001	0,614

Note: Fisher's exact test output: Significance by cell indicates if the actual value is equal (=), lower (<) or higher (>) than the theoretical value; Values displayed in red are significant: $p \leq 0.05$; Grey highlight: actual value higher (>) than theoretical

Table S10. Meal confirmations by user group

	General public	People that have overweight	People with obesity	Athletes & exercisers
Increase body weight	>	<	<	>
Maintain body weight	>	<	<	>
Increase muscle mass	>	<	<	<
Increase iron intake	>	<	<	>
Decrease fat intake	>	<	>	<
Increase fruit intake	>	<	<	<
Increase vitamin D intake	>	>	<	<
Increase vegetable intake	>	<	<	<
Increase activity intensity	<	<	<	>
Increase fluid intake	>	>	<	>
Increase activity duration	>	<	>	<
Decrease body fat	>	<	>	<
Decrease body weight	<	>	>	<

	General public	People that have overweight	People with obesity	Athletes & exercisers
Increase body weight	<0,0001	<0,0001	<0,0001	<0,0001
Maintain body weight	0,299	<0,0001	<0,0001	<0,0001
Increase muscle mass	<0,0001	<0,0001	<0,0001	<0,0001
Increase iron intake	<0,0001	<0,0001	0,109	0,013
Decrease fat intake	<0,0001	<0,0001	0,576	<0,0001
Increase fruit intake	<0,0001	<0,0001	<0,0001	<0,0001
Increase vitamin D intake	<0,0001	0,100	0,007	<0,0001
Increase vegetable intake	<0,0001	<0,0001	<0,0001	<0,0001
Increase activity intensity	<0,0001	0,056	<0,0001	<0,0001
Increase fluid intake	0,227	0,096	<0,0001	0,000
Increase activity duration	<0,0001	<0,0001	0,011	<0,0001
Decrease body fat	<0,0001	0,004	0,741	<0,0001
Decrease body weight	<0,0001	<0,0001	<0,0001	<0,0001

Note: Fisher's exact test output: Significance by cell indicates if the actual value is equal (=), lower (<) or higher (>) than the theoretical value; Values displayed in red are significant: $p \leq 0.05$; Grey highlight: actual value higher (>) than theoretical
